# Genomic characterization of functional high-risk multiple myeloma patients

**DOI:** 10.1038/s41408-021-00576-3

**Published:** 2022-01-31

**Authors:** Cinnie Yentia Soekojo, Tae-Hoon Chung, Muhammad Shaheryar Furqan, Wee Joo Chng

**Affiliations:** 1 Department of Hematology-Oncology, National University Cancer Institute, National University Health System, Singapore, Singapore; 2 Cancer Science Institute of Singapore, National University of Singapore, Singapore, Singapore

**Keywords:** Cancer genomics, Myeloma

## Abstract

Multiple myeloma (MM) patients with suboptimal response to induction therapy or early relapse, classified as the functional high-risk (FHR) patients, have been shown to have poor outcomes. We evaluated newly-diagnosed MM patients in the CoMMpass dataset and divided them into three groups: genomic high-risk (GHR) group for patients with t(4;14) or t(14;16) or complete loss of functional TP53 (bi-allelic deletion of TP53 or mono-allelic deletion of 17p13 (del17p13) and TP53 mutation) or 1q21 gain and International Staging System (ISS) stage 3; FHR group for patients who had no markers of GHR group but were refractory to induction therapy or had early relapse within 12 months; and standard-risk (SR) group for patients who did not fulfill any of the criteria for GHR or FHR. FHR patients had the worst survival. FHR patients are characterized by increased mutations affecting the IL-6/JAK/STAT3 pathway, and a gene expression profile associated with aberrant mitosis and DNA damage response. This is also corroborated by the association with the mutational signature associated with abnormal DNA damage response. We have also developed a machine learning based classifier that can identify most of these patients at diagnosis.

## Introduction

There is an increasing appreciation that risk stratification is important in the management of multiple myeloma (MM) [1]. Despite advancement of MM treatment in the past decades following the introduction of proteasome inhibitors and immunomodulatory drugs, which, together with autologous stem cell transplantation (ASCT), have improved the median overall survival from 3 to 8 years [2–4], there are still ~20% of patients who survive for only ~3 years. Improving the outcome of these high-risk patients is one of the most important current therapeutic challenge in MM.

The current approach to risk stratification uses genetic information from FISH, with t(4;14) or t(14;16) or del17p13 by FISH identified as high-risk genetic abnormalities, as well as clinical information such as serum albumin and beta-2 microglobulin and lactate dehydrogenase (LDH), in the R-ISS staging system [5]. Recently, a specific entity named the double-hit MM with del17p13 and TP53 mutation or 1q amplification (four or more copies) and International Staging System (ISS) stage 3 has been shown to have poor survival with median progression-free survival (PFS) of 15.4 months and median overall survival (OS) of 20.7 months [6]. Many published and ongoing studies have also evaluated MM gene expression signatures such as EMC92 [7], GEP70 [8] to identify high-risk patients, although this has not been routinely used in clinical practice. Some clinical characteristics such as extramedullary plasmacytomas [9], presence of circulating tumor cells [10] and renal failure [11, 12] have also been associated with high-risk disease, but they have not been consistently included as criteria for clinical trials.

Recently, a number of studies have highlighted that multiple myeloma patients with suboptimal response to induction therapy or early relapse have been shown to have poor outcomes [13, 14].

A real-world outcome study of 1320 newly diagnosed patients by Australian and New Zealand Myeloma and Related Diseases Registry (MRDR) showed that 40% of patients with suboptimal response to induction therapy died within 3 years of diagnosis, and patients who had early disease progression within 12 months of starting induction therapy had median OS of only 20.2 months [13]. These patients are categorized as the functional high-risk (FHR) MM patients. However, it is not clear how many of these patients also have high-risk genetic features.

In this study, we assess the criteria of FHR patients with a more refined definition including only those who either have suboptimal response to therapy or progress within 12 months of starting induction therapy but yet do not have any of the clinically applicable high-risk genetic features. In addition, we comprehensively compare their genomic profiles (DNA mutations, mutational signatures (MS), transcriptional signatures, copy number abnormalities) with other MM patients to gain insights into what may drive this phenotype of extremely poor outcome.

## Methods

We evaluated genomic sequencing and high-throughput molecular assay data of newly diagnosed MM patients in the CoMMpass dataset (IA13a version), a publicly available dataset from the Multiple Myeloma Research Foundation (MMRF).

We divided the patients into three groups: genomic high-risk (GHR) group for patients with t(4;14) or t(14;16) or complete loss of functional TP53 (bi-allelic deletion of TP53 or mono-allelic deletion of 17p13 (del17p13) and TP53 mutation) or 1q21 gain and International Staging System (ISS) stage 3; FHR group for patients who had no markers of GHR group but were refractory to induction therapy or had early relapse within 12 months; and standard-risk (SR) group for patients who did not fulfill any of the criteria for GHR or FHR.

To categorize the patients into their respective risk groups, we used the following CoMMpass data: translocations of IGH locus and their partners using RNA-seq data for canonical Ig translocation, copy number aberrations (CNAs) using CNA segmentation data, response to the first line therapy using per patient treatment response data, ISS staging information using per patient aggregate of clinical information, and disease progression information using per patient survival data.

We analyzed the transcriptome data to obtain clues of underlying molecular mechanisms driving FHR or GHR. We first identified differentially expressed genes (DEGs) using SAM [15] for multiple groups and then queried top DEGs to DAVID [16] to infer their functional consequences. To account for the distortion introduced during the selection of top DEGs, we also employed gene set enrichment analysis (GSEA; v4.1) [17] and captured transcriptomic changes in the genome-wide scale. We also used the following gene expression signatures to investigate the utility of these signature indices in identifying FHR patients based on transcriptomic data: centrosome index (CI) [18], chromosomal instability (CIN) index by Carter et al. (CIN70) [19], CIN index from sarcoma study (CINSARC) [20], CIN index of our own (CINGEC) [21], 92-gene survival index from HOVON-65/GMMG-HD4 study (EMC92) [7], 7-gene survival index from an MM cell line study (HMCL7) [22], a signature of cell death genes affected by homozygous deletion (HZDCD) [23], 15-gene survival index from Intergroupe Francophone du Myeloma study (IFM15) [24], proliferation index (PI) [25], a gene signature index proliferation associated genes from HOVON-65/GMMG-HD4 study (PR) [7], 70-gene and 80-gene survival index from University of Arkansas Medical School (UAMS70 [8], UAMS80 [26]).

We evaluated the copy number aberrations (CNAs) to uncover CNA features associated with FHR or GHR. Starting from the segmentation data provided in the CoMMpass data portal, we determined optimal threshold values for different CNA status as well as minimal segment size to retain and applied them to the segmentation data to determine CNA status for each segment. Subsequently, CNA status was transformed into a matrix format (Supplementary Fig. 1). The CIN index [21] we had developed before was also employed to see if there could be any difference in CIN and hence the biological mechanism to ensure chromosomal integrity among different risk groups. Mutation status of each patient was also analyzed to identify genes and pathways that were preferentially mutated among risk groups using non-synonymous (NS) mutation data compiled in CoMMpass. MS were also assessed and examined for difference in propensity among risk groups using tools and data compiled in COSMIC (v3.2) [27].

To build a predictive model to identify FHR patients using machine learning, we generated six sets of data for each patient based on various features: the number of mutations for each of 15,633 genes that harbored at least one NS mutation (mutation_matrix; 15,633 features), the number of all NS mutations for each of 44 chromosomal arms (mutation_count_by_arm; 44 features), the CNA status for each of 13,155 genes that harbored at least one CNA (cna_by_gene_reduced; 13,155 features), the CNA status for each of 44 chromosomal arms (cna_by_arm; 44 features), transcriptome data of 25,554 genes from RNA-seq where each gene’s expression profile was normalized against its median level (gep_normed; 25,554 features), and six parameters including age, gender, creatinine level, ECOG status, ISS staging, and proliferation index (clinical_parameters; 6 features). In determining cna_by_gene_reduced and cna_by_arm, if multiple CNA statuses appear in a gene or a chromosomal arm, we selected the CNA status of dominant span in a gene or a chromosomal arm. The details of these features are listed in the Supplementary Table 1 and Supplementary File 1.

We undertook the following pre-processing of the six datasets. We first removed highly correlated features from each dataset to reduce the overall feature space and to concentrate on the features that had more meaningful information. We used an absolute correlation threshold of 0.5, and, for some datasets, we performed a significance test to further limit the feature space. In training and testing machine learning models, each dataset was divided into two subsets using a 70–30 rule where 70% of the data were used for training models whereas the remaining 30% were used for testing. During data division, we took a special effort to maintain the distribution of positive and negative cases in both testing and training datasets.

As CoMMpass data is greatly affected by severe class imbalance, we employed a widely used oversampling technique called Synthetic Minority Oversampling Technique (SMOTE) [28] to reduce the bias towards the majority class during the modeling stage. SMOTE creates synthetic minority class samples using KNN technique and potentially performs better than simple oversampling [29]. It has been used in several studies such as breast cancer detection [30], miRNA gene prediction [31, 32], and for the identification of the binding specificity of the regulatory proteins [33].

We used the random forest algorithm for predicting FHR cases in this study. Random forest is an ensemble decision tree-based technique where each tree registers a vote for the most prevalent class and the final decision is made on consensus majority votes [34]. Despite its algorithmic simplicity, it is known to perform fairly well and has been widely used in bioinformatics e.g. for the classification of mRNA microarray data [35], to detect biomarkers for prostate cancer progression [36] and more. The performance of prediction results on test datasets were evaluated for all six individual models on multiple measures such as specificity, sensitivity, false-negative rate (FNR), False positive rate (FPR), accuracy, F1-Score, and Matthews correlation coefficient (MCC) (Supplementary Fig. 2).

## Results

### FHR patients had the worst survival

Of the 512 evaluable patients, there were 345 patients in the SR group, 106 patients in the GHR group, and 61 patients in the FHR group. The available baseline clinical characteristics of these patients were listed in Table 1. As expected, there are more ISS III and Revised ISS III patients in the GHR group. Interestingly, there are no unique clinical characteristics to FHR patients, in particular, very few are R-ISS III. Most patients had proteasome inhibitor (PI) and immunomodulatory (IMiD) drug combination as first line treatment, while a smaller proportion of patients had PI-based or IMiD-based treatment. The treatment received across the three groups were similar.

**Table 1:** Baseline characteristics.

		SR (*N* = 345) (%)	GHR (*N* = 106) (%)	FHR (*N* = 61) (%)
Gender	Male	186 (53.9)	67 (63.2)	39 (63.9)
	Female	159 (46.1)	39 (36.8)	22 (36.1)
Age	Median (year)	62	61	65
ISS	Stage I	142 (41.2)	31 (29.2)	17 (27.9)
	Stage II	115 (33.3)	42 (39.6)	20 (32.8)
	Stage III	88 (25.5)	33 (31.1)	24 (39.3)
Revised-ISS	Stage I	111 (32.2)	1 (0.9)	13 (21.3)
	Stage II	211 (61.2)	74 (69.8)	43 (70.5)
	Stage III	23 (6.7)	31 (29.2)	5 (8.2)
Treatment class	PI/IMiD combination-based	367 (48.1)	99 (46)	50 (42.7)
	PI-based	233 (30.5)	68 (31.6)	43 (36.8)
	IMiD-based	126 (16.5)	39 (18.1)	18 (15.4)
	Others	37 (4.8)	9 (4.2)	6 (5.1)

*SR* Standard Risk group, *GHR* Genomic High-Risk group, *FHR* Functional High-Risk group, *ISS* International Staging System, *PI* Proteasome Inhibitor, *IMiD* Immunomodulatory Drug.

Data are number (%).

**Fig. 1:**
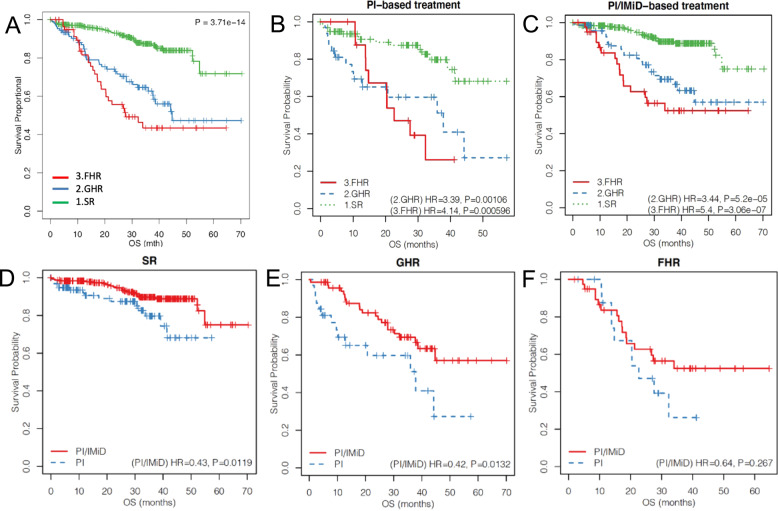
Survival curves for FHR, GHR, and SR MM patients in CoMMpass dataset. **A** Overall (**B**) FPI-based induction treatment and (**C**) PI/IMiD-based induction treatment. Survival curves for patients treated with PI and PI/IMiD in (**D**) SR, (**E**) GHR, (**F**) FHR groups, respectively. *P*-values indicate Cox regression test of PI/IMiD treatment against PI treatment.

On the survival analysis, both FHR and GHR groups had significantly poorer outcomes compared to the SR group, with FHR group being the worst. The median OS for the FHR group was 27.6 months, while the median OS was 44.7 months for the GHR group, and not reached for the SR group (FHR: HR = 5.19, *p* = 3.42 × 10^−11^; GHR: HR = 3.55, *p* = 3.5 × 10^−8^) (Fig. 1A). Similar patterns were seen for these group of patients when treated with PI-based and PI/IMiD combination induction therapies (Fig. 1B–F).

### FHR patients cannot be easily identified using known high-risk gene expression signatures or combination of high-risk genetic features

**Fig. 2:**
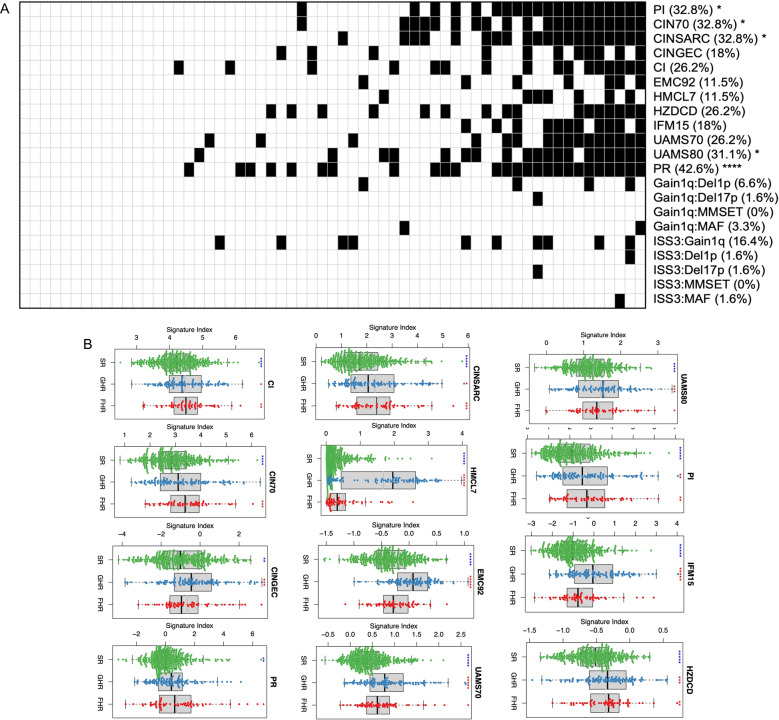
FHR MM patients and known high-risk signatures. **A** Each column indicates individual FHR patient. For gene expression signatures—PI, CIN70, CINSARC, CINGEC, CI, EMC92, HMCL7, HZDCD, IFM15, UAMS70, UAMS80, PR—we arbitrarily set patients with top 20% signature indices as high risk (black square). For other combination high-risk genetic markers—Gain1q:Del1p, Gain1q:Del17p, Gain1q:MMSET, Gain1q:MAF, ISS3:Gain1q, ISS3:Del1p, ISS3:Del17p, ISS3:MMSET, ISS3:MAF—the presence of such combination markers was indicated (black square). Individual genetic markers are as follow: gain1q (gain of 1q), del1p (deletion of 1p), del17p (deletion of 17p), MMSET (dysregulation of MMSET), MAF (dysregulation of MAF/MAFB/MAFC), and ISS3 (ISS stage 3). **B** Gene expression signature box plots. Symbols above each risk group indicate statistical significance (*: 0.01 < *p* ≤ 0.05; **: 10^−3^ < *p* ≤ 10^−2^; ***: 10^−4^ < *p* ≤ 10^−3^; ****: 10^−5^ < *p* ≤ 10^−4^; *****: *p* ≤ 10^−5^) of comparison between a specific group and all the rest. Symbol colors indicate whether the mean level of a specific group is above (red) or below (blue) that of all the rest.

We applied established gene expression signatures of high-risk disease, including proliferation (PI), chromosomal instability (CIN70, CINSARC, CINGEC), centrosome (CI), cell death (HZDCD), and others (EMC92, HMCL7, IFM15, UAMS70, and UAMS80), to see if these FHR cases are characterized by them. Interestingly, most of the FHR patients do not have these high-risk gene expression signature (Fig. 2A). In fact, when compared across the three groups (SR, GHR, and FHR), these signatures are not significantly different between SR and FHR (HMCL7, UAMS80, UAMS70, EMC92, IFM15, and CINGEC) or between GHR and FHR (PI, HZDCD, CINSARC, CI, CIN70, and PR), although the indices associated with chromosomal instability (CINSARC, CI, and CIN70) or tumor aggression (PI and PR) appear to be generally higher in the FHR patients (Fig. 2B). We also used several different combinations of high-risk features including the PR index, gain1q21 + del1p, gain1q21 + del17p13, gain1q21+MMSET, gain1q21+MAF, ISS3 + gain1q21, ISS3 + del1p, ISS3 + del17p13, ISS3 + MMSET, and ISS3 + MAF, to evaluate if they could identify these FHR patients. These combinations of markers are rarely present in FHR patients (Fig. 2A).

This suggests that the FHR patients are generally not characterized by known high-risk signatures that have been described.

### What are the genomic features of FHR MM patients?

We next explored the mutational, transcriptional and copy number landscape of these patients to see if unique molecular and genomic abnormalities can be identified.

#### Mutation analysis

**Fig. 3:**
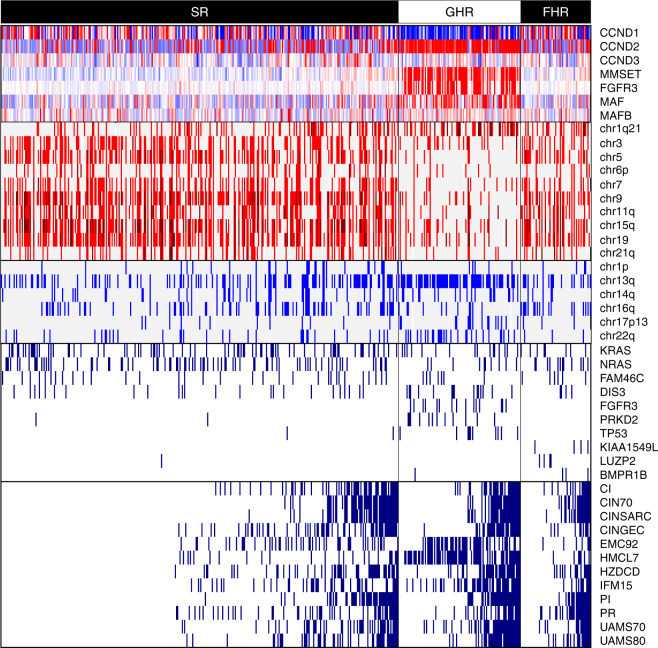
Composite heat map combining gene expression, copy number aberration, mutation, and gene expression signatures. Top panel (CCND1, CCND2, CCND3, MMSET, FGFR3, MAF, MAFB) shows the median-normalized gene expression profiles of important TC class marker genes. For each gene, expression above/below the median level is indicated as red/blue, and the median level is indicated as white. The second panel comprises chr1q21, chr3, chr5, chr6p, chr7, chr9, chr11q, chr15q, chr19, chr21q which displays gain of the respective chromosomal regions. Single copy gain is indicated as red and two or more copy gain is indicated as dark red. The GHR group clearly shows the dominance of non-hyperdiploid cases while the SR and FHR groups show prevalence of hyperdiploid cases. The third panel comprises chr1p, chr13q, chr14q, chr16q, chr17p13, chr22q which exhibits loss of respective chromosomal regions. Single copy loss is indicated as blue and two-copy loss is indicated as dark blue. The GHR group clearly shows the dominance of chr13q deletion, possibly indicating the involvement of RB1. The fourth panel comprises KRAS, NRAS, FAM46C, DIS3, FGFR3, PRKD2, TP53, KIAA1549L, LUZP2, and BMPR1B which shows presence of NS mutations for the respective genes. KRAS, NRAS, FAM46C, and DIS3 are known to be frequently mutated in MM. However, FGFR3, PRKD2, and TP53 genes are found to be mutated specifically in the GHR group, while KIAA1549L, LUZP2, and BMPR1B genes are found to be mutated specifically in the FHR group in this study. The bottom panel comprises CI, CIN70, CINSARC, CINGEC, EMC92, HMCL7, HZDCD, IFM15, PI, PR, UAMS70, and UAMS80 which represents gene expression signatures. Here, patients with top 20% respective indices are marked as high-risk.

We analyzed the NS mutations in the CoMMpass data and evaluated the prevalence of mutation for genes known to be frequently mutated in MM (KRAS, NRAS, and FAM46C) between different risk groups using the Fisher’s exact test. There was no obvious concentration of mutations in any risk group. However, we uncovered that the GHR group had higher mutational load (*p* = 0.00331), and genes such as FGFR3 (*p* = 1.63 × 10^−11^), PRKD2 (*p* = 2.82 × 10^−7^), and TP53 (*p* = 8.7 × 10^−6^) were predominantly mutated in GHR group as compared with others. On the other hand, KIAA1549L, LUZP2, and BMPR1B were predominantly mutated in FHR (Fig. 3).

We then assessed whether the mutated genes were enriched for any gene sets in each risk group, and analyzed the differences with Wilcoxon’s rank-sum test. In the FHR group, the IL6-JAK-STAT3 pathway was found to be significantly enriched (*p* = 0.00924), while estrogen response (*p* = 0.000369), KRAS (*p* = 0.000417), and WNT β catenin (*p* = 0.000447) signaling pathways were found to be enriched in the GHR group.

#### Copy number aberrations

Of the 471 evaluable patients, 224 (47.6%) were non-hyperdiploid and 247 (52.4%) were hyperdiploid. Interestingly, FHR group was predominantly hyperdiploid (57.9% vs 42.1% non-hyperdiploid) while GHR group was mostly non-hyperdiploid (90.8% vs 9.2% hyperdiploid). GHR group also had more pronounced 13q deletion, and increased 1q21 gain compared to FHR (*p* = 1.45 × 10^−10^) and SR groups (*p* < 2.2 × 10^−16^). The SR group had more hyperdiploid (64.9% vs 35.1% non-hyperdiploid) patients, which was statistically insignificant compared to the FHR group (*p* = 0.369) (Fig. 3 and Supplementary Fig. 3). Therefore, the copy number profile of FHR patients were similar to that of SR patients.

We also estimated CIN using CNAs over autosomal chromosomes. There were no statistically significant differences between FHR and SR groups (*p* = 0.194) or between GHR and SR groups (*p* = 0.516), or between FHR and GHR groups (*p* =0.427) (Supplementary Fig. 4).

#### Differentially expressed genes and enriched pathways

Using the RNA-seq data, we evaluated the DEGs. The DEGs in FHR and GHR groups were distinct. The list of DEGs from SAM are listed in Supplementary File 2.

In order to understand the biological processes implicated by the list of DEGs, we queried the functional annotations using DAVID for top 200 genes from the comparison between FHR and SR groups but excluding those turned out to be significant from the comparison between GHR and SR groups (Supplementary File 3). FHR patients were found to be enriched for genes linked to mitotic cell cycle and DNA replication, C2H2 zinc finger, and DNA repair. For instance, the first, second, and several of the top sixth and eighth annotation clusters were composed of highly significant terms such as centromere, mitotic cell cycle, and DNA replication, displaying the significant association of mitotic cell cycle processes to FHR. The third annotation cluster was composed of mostly C2H2 zinc finger related terms that are rather too generic to infer further biological context directly. However, a recent publication [37] links the recruitment of C2H2 zinc finger domain to cereblon (CRBN) and the induction of the ubiquitination and proteasomal degradation of genes targeted by small molecules thalidomide and its analogs, lenalidomide and pomalidomide, thereby suggesting potential relevance of this cluster. The fourth, fifth, and top of seventh annotation clusters were associated with DNA repair.

We also queried the functional annotations using the top 499 genes from the comparison of GHR and SR but excluding those significant between FHR and SR groups (Supplementary File 4) to DAVID. GHR patients were enriched for genes linked to ribosomal RNA/protein and protein translation initiation and Ig subtype clusters.

To mitigate the limitation of functional annotations due to artificial selection of top DEGs, we additionally performed GSEA. Compared to the SR group, FHR group showed enrichment in a number of gene sets of the hallmark group known to be involved in MM (*P* < 0.05 & FDR < 0.25), including E2F Targets, G2M Checkpoint, MTORC1 signaling, Glycolysis, Unfolded protein response, Myc targets, DNA repair, while no significant gene set enrichment were found for the SR group (Table 2). The full list of GSEA result is shown on the Supplementary File 5. For the GHR group, five gene sets were found enriched (Androgen response, Estrogen response, Glycolysis, UV response, and IL2-STAT5 signaling) as compared to the SR group (Table 3). The full list is shown on the Supplementary File 6.

**Table 2:** Top hallmark gene sets (P < 0.05 & FDR < 0.25) enriched in FHR group.

Term	ES	NES	*P*-value	FDR	Size
Hallmark_E2F_Targets	0.72	1.77	0.00412	0.165	197
Hallmark_G2M_Checkpoint	0.65	1.74	0.01030	0.118	195
Hallmark_MTORC1_Signaling	0.54	1.70	0.00795	0.110	196
Hallmark_Glycolysis	0.45	1.66	0.00877	0.124	198
Hallmark_Unfolded_Protein_Response	0.57	1.66	0.01170	0.100	109
Hallmark_MYC_Targets_V1	0.62	1.59	0.02760	0.141	194
Hallmark_Bile_Acid_Metabolism	0.42	1.54	0.00952	0.171	110
Hallmark_Fatty_Acid_Metabolism	0.45	1.54	0.02650	0.158	156
Hallmark_DNA_Repair	0.54	1.51	0.02330	0.171	146
Hallmark_Peroxisome	0.44	1.47	0.03650	0.203	104
Hallmark_Estrogen_Response_Late	0.33	1.40	0.01760	0.205	198

*ES* Enrichment Score, *NES* Normalized Enrichment Score, *FDR* False Discovery Rate.

**Table 3:** Top hallmark gene sets (*P* < 0.05 and FDR < 0.25) enriched in GHR group.

Term	ES	NES	*P*-value	FDR	Size
Hallmark_Androgen_Response	0.46	1.64	0.01300	0.233	98
Hallmark_Estrogen_Response_Early	0.39	1.64	0.00515	0.160	198
Hallmark_Glycolysis	0.41	1.49	0.04490	0.218	198
Hallmark_UV_Response_DN	0.37	1.43	0.04280	0.226	142
Hallmark_IL2_STAT5_Signaling	0.35	1.43	0.04950	0.207	198

*ES* Enrichment Score, *NES* Normalized Enrichment Score, *FDR* False Discovery Rate.

#### Mutational signatures

**Fig. 4:**
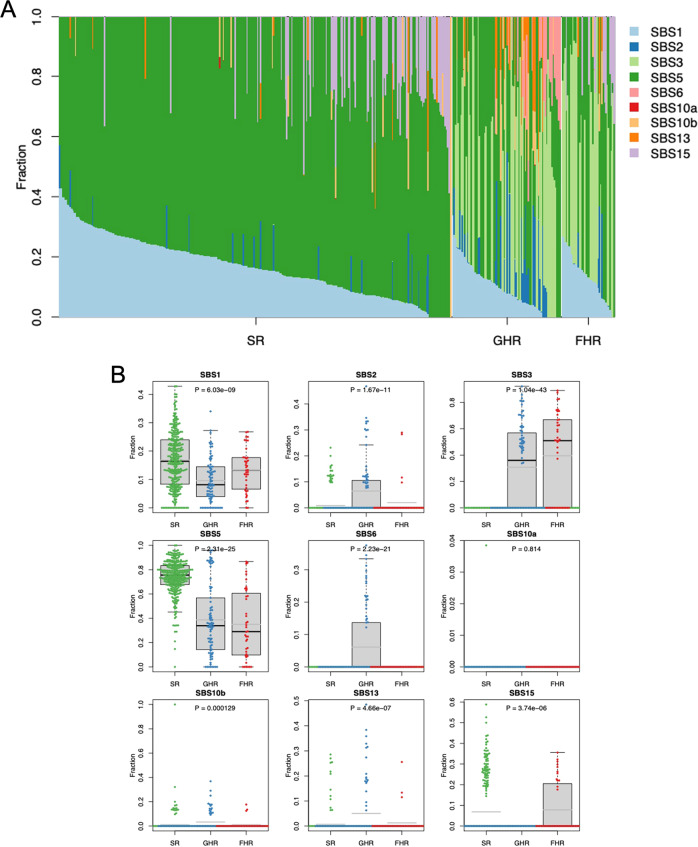
Mutational Signatures for SR, GHR, and FHR groups. **A** For each patient, respective contributions of component MSs are indicated with different color codes. **B** For individual MSs, respective level of contributions among all MSs per patient are compared among the three risk groups using Kruskal-Wallis test. Here, whether the distribution of respective level of contributions in one risk group is particularly different from those of the other risk groups is tested. SBS5 is more prominent in the SR group than in the GHR and FHR groups (*p* = 2.31 × 10^−25^), while SBS3 is more prominent in the GHR and FHR groups (*p* = 1.04 × 10^−43^) . SBS2 (*p* = 1.67 × 10^−11^) and SBS6 (*p* = 2.23 × 10^−21^) are more prominent in the GHR group.

We also analyzed the MS using the SigProfiler with Catalogue Of Somatic Mutations in Cancer (COSMIC) reference catalogue [38]. SBS1 and SBS5 were highly specific to SR. SBS3 was highly specific to the GHR and FHR groups. SBS6 was very specific to GHR (Fig. 4**)**. There was therefore no MS specific only to FHR.

### Machine learning based predictor for FHR patients

It is clear that none of the current signatures or high-risk features can identify majority of these FHR patients very well. We therefore used machine learning methodology to develop a classifier.

Our machine learning based predictor showed that performance of the individual model was not optimal (Supplementary Table 2). In order to improve the model and to obtain results that are more robust and useful, we decided to stack multiple models (Supplementary Table 3).

For the highest accuracy, a model with the combination of mutation matrix, gep_normed and clinical parameters, with an accuracy of 0.75, specificity of 0.76, sensitivity of 0.67, false negative rate of 0.33, false positive rate of 0.23, Area Under the Receiver Operating Curve (AUC-ROC) of 0.71, F1 score of 0.34, and MCC score of 0.28, can be used (Supplementary Fig. 5). To ensure that we do not give unnecessary treatment to non-FHR patients and avoid harm, we may want use the model with highest specificity with lowest false positive rate with a combination of cna_by_gene_reduced, mutation_matrix, gep_normed, and clinical parameters, which has an accuracy of 0.78, specificity of 0.80, sensitivity of 0.60, false negative rate of 0.4, false positive rate of 0.2, AUC-ROC of 0.70, F1 score of 0.35, and MCC score of 0.28. However, if the treatment proposed for the FHR patients is not likely to increase harm and can also benefit non-FHR patients, we may want to use a model that gives maximum sensitivity with lowest false negative rate, using a combination of gep_normed and mutation matrix. This model has an accuracy of 0.65, specificity of 0.63, sensitivity of 0.87, false negative rate of 0.13, false positive rate of 0.37, AUC-ROC of 0.75, F1 score of 0.33, and MCC score of 0.30.

## Discussion

In this study, we showed that the FHR MM patients that do not have any of the known clinically applied high-risk genetic factors have very poor outcomes. Most of these patients also do not harbor other high-risk characteristics that have been published. As these FHR MM patients are defined based on poor response to induction treatment and early disease progression, there is currently no easy way to identify them at diagnosis. There is much interest to design clinical trials specifically targeting high-risk patients as they need a different therapeutic strategy. However, our current definition of high-risk would have failed to identify these FHR patients. In this study, we developed a machine learning classifier that allows us to identify FHR patients a priori.

Understanding the genomics and biology of these FHR patients may also provide insights into potential therapeutic strategy. New therapeutic approaches are needed for these patients as current approaches have not improved their outcomes significantly. FHR patients seem to have increased mutations affecting the IL-6/JAK/STAT3 signaling pathway. The IL-6/JAK/STAT3 signaling has been shown to drive the proliferation, survival, invasiveness, and metastasis of cancer cells, while suppressing the antitumor immune response [39]. The IL-6/JAK/STAT pathway is also important in myeloma and may be a good therapeutic target in myeloma [40]. We have previously shown that IL6-STAT activation may drive high-risk phenotypes via promotion of aberrant RNA editing through upregulation of ADAR1 [41] and also upregulation of a high-risk phosphatase, PRL-3 [42]. Some studies showed that MM cells with an IL-6-activated JAK/STAT3 pathway are particularly sensitive to heat shock protein 90 (Hsp90) inibitors [43], making this a potential therapeutic target for the FHR MM patients.

The DEG and MS point to the importance of genomic instability, aberrant centromere, mitosis, and abnormal DNA damage repair. Chromosome instability has been known to be a hallmark in MM [44]. Centromeres and their associated kinetochores play an important role in affecting cell mitosis and therefore chromosomal integrity [45]. Along with the context of mitosis, centrosome amplification has also been shown to have prognostic implication in MM. A gene expression-based centrosome index (CI) of more than 4, which was calculated by adding the normalized expression value of the expression levels of genes encoding for the proteins in the centrosomes, has been previously shown to be associated with short survival in MM [46]. The Intergroupe Francophone du Myelome (IFM) from France showed that overexpression of genes involved in mitosis was associated with high-risk disease resulting in poor survival [24]. Similarly, a study from the United Kingdom showed that mutations in the DNA damage pathways are associated with poor outcomes [47]. Recently, we have showed that NEIL1, a gene involved in DNA damage repair, is hyperedited in MM patients with poor outcome and leads to aberrant DNA damage response in these cells [48]. Synthetic lethal approach to exploit DNA damage repair abnormalities in MM has been studied, showing an addiction in these cells to ATR inhibition [49, 50]. In our recent studies, we showed that targeting CHEK1 is also a synthetic lethal approach in high-risk disease with abnormal DNA damage repair phenotype [51].

However, what is most striking is how little distinguished FHR patients are from GHR and SR patients genomically in terms of transcriptomics, MS, copy number, and genes affected by somatic mutations. The lack of distinguishing genomic profile in FHR patients might also suggest that factors outside of the tumor cells such as the immune dysregulation or tumor microenvironment may play an important role in FHR patients. This will need to be addressed in future studies. There are two important implications. First, understanding the differences outside of the tumor cells may allow the development of more effective strategies against the FHR patients as current therapies targeting the traditional myeloma vulnerabilities are ineffective. Second, while our current artificial intelligence (AI) model is better than existing tools in identifying FHR patients, the additional knowledge about the immune dysregulation and tumor microenvironment may add to the model to make the model even better or in fact may simplify the model if the differentiating power is greater.

In summary, we have shown that FHR MM patients, even without any high-risk genetic factors, have very poor outcomes, and we developed a machine learning based classifier that can identify most of these patients at diagnosis. These patients are characterized by increased mutations affecting the IL-6/JAK/STAT3 pathway, and a gene expression profile associated with aberrant mitosis and DNA damage response. This is also corroborated by the association with the MS associated with abnormal DNA damage response. Targeting the STAT pathway and taking advantage of synthetic lethal addictions to the abnormal DNA damage response may lead to novel therapeutic strategies to explore for these patients.

### Reporting summary

Further information on research design is available in the Nature Research Reporting Summary linked to this article.

## Supplementary information

Reporting summary checklist

Supplementary figures, tables and details of supplementary files

Supplementary File 1

Supplementary File 2

Supplementary File 3

Supplementary File 4

Supplementary File 5

Supplementary File 6
