# Supplementary material for: Genomic characterization of functional high-risk multiple myeloma patients
Source: Blood Cancer J. 2022 Jan 31;12(1):24. doi: 10.1038/s41408-021-00576-3 (PMC8803925; doi:10.1038/s41408-021-00576-3)
Supplement: Supplementary file 2 — Supplementary figures, tables and details of supplementary files [file 41408_2021_576_MOESM2_ESM.pdf]

Supplementary Figures

Supplementary Figure 1. Determination of optimal cut-off values of CNA status and minimal segment size.

The distribution of segments' mean log-ratio (MLR) values without any filtering:

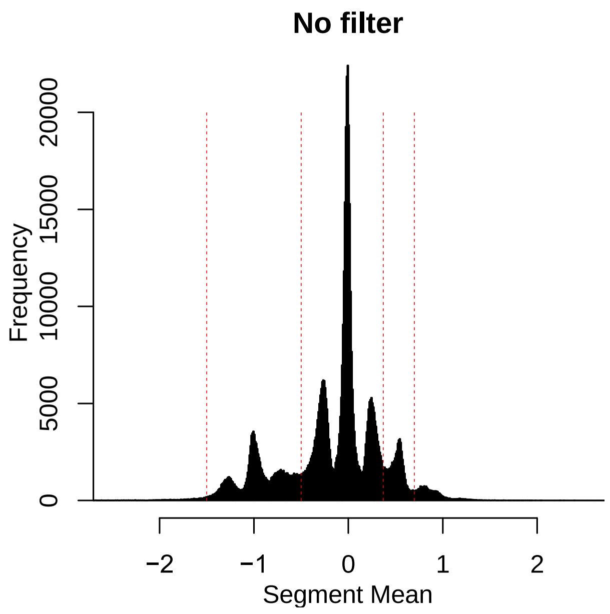

After restriction of segments with lengths ≥ 1 000 probes and retention of reliable segments:

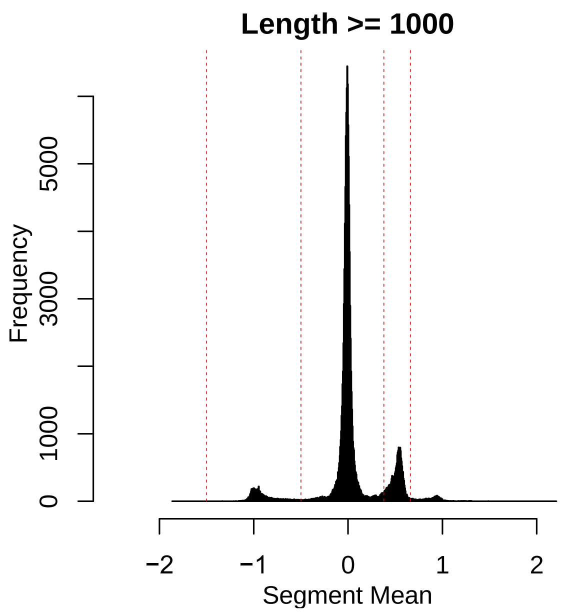

Based on the above, the peaks in the unfiltered distribution might be spurious, and filtering based on segment length can aid in our selection. We then evaluated a series of segment length filters to determine the appropriate segment length threshold as well as the MLR cutoff values to delineate different CNA statuses.

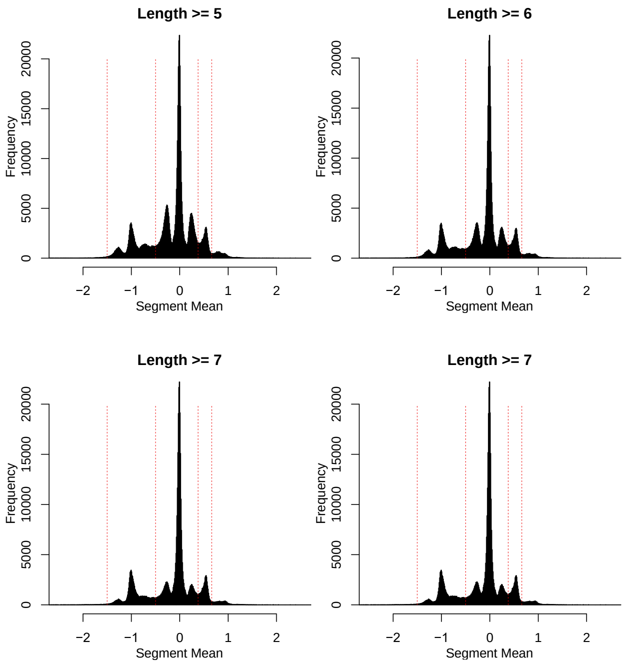

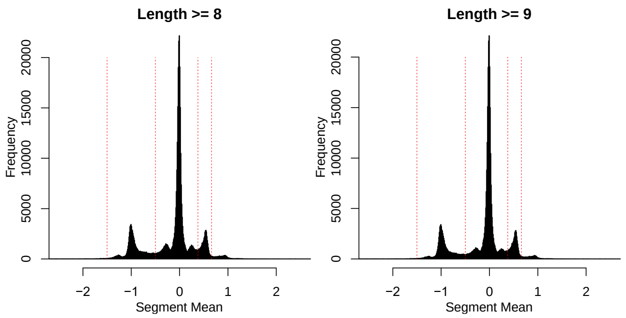
Length >= 8
Length >= 9
Frequency
0
5000
10000
15000
20000
−2
−1
0
1
2
Segment Mean

18

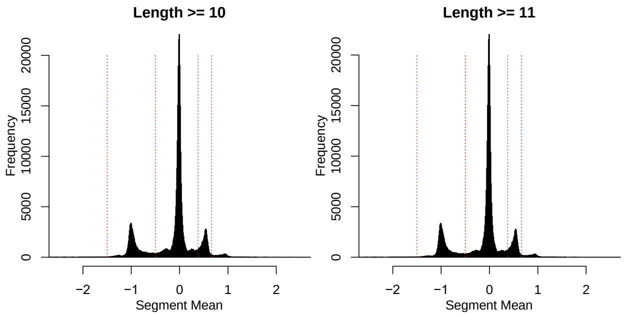
Length >= 10
Length >= 11
Frequency
0
5000
10000
15000
20000
−2
−1
0
1
2
Segment Mean

19

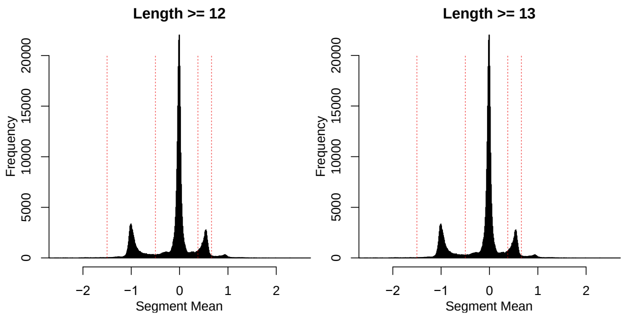
Length >= 12
Length >= 13
Frequency
0
5000
10000
15000
20000
−2
−1
0
1
2
Segment Mean

20

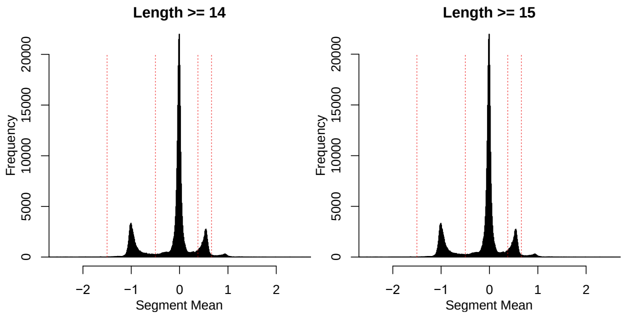
Length >= 14
Length >= 15
Frequency
0
5000
10000
15000
20000
−2
−1
0
1
2
Segment Mean

21

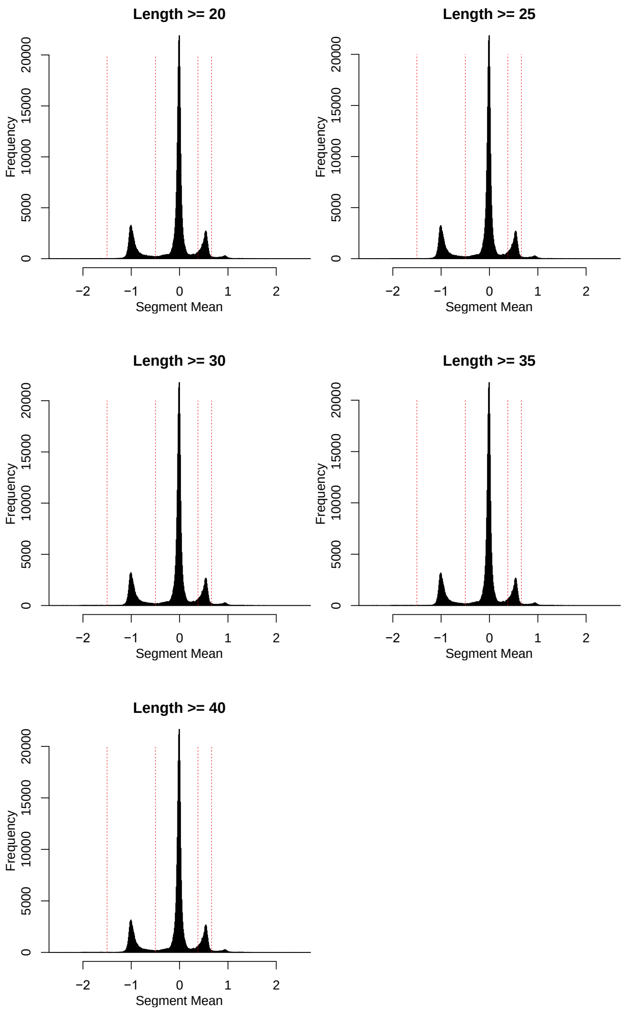

Histograms for length threshold of 15 probes and beyond was similar with the histogram with length threshold of 1 000 probes. With appropriate MLR cut-off values, we might still able to lower the segment length threshold further down and delineate different CNA statuses as we might have achieved with the most reliable long length segments only, even in the presence of traces of spurious peaks. Therefore, in this analysis, we used segment length threshold of 10 probes and MLR cutoff values of -1.5, -0.5, 0.38, 0.66 (red vertical lines in the histograms). Consequently, segments

of lengths ≥ 10 probes were assigned the following CNA status depending on their MLR values:

- -2 if MLR ≤ -1.5
- -1 if -1.5 < MLR ≤ -0.5
- 0 if -0.5 < MLR ≤ 0.38
- +1 if 0.38 < MLR ≤ 0.66
- +2 if MLR > 0.66

We transformed the CNA information in the form of segmentation data into a matrix form where rows were small spans of genome and columns were patients. The CNA status of a patient in a given genomic span could then be easily identified by referencing the proper element of the CNA matrix.

The transformation from the segmentation format to the matrix format was proceeded in a chromosome-by-chromosome manner. For each chromosome (1, …, 22, X, Y), we gathered all unique starting positions and the last end position of segments. When ordered in an ascending order, these positions serve as natural delineating boundaries of the chromosome. We call these small spans delineated by unique starting positions of segments as blocks. Subsequently, the value of (*m*, *n*)-th element of the CNA matrix could be assigned by the predefined CNA level of a segment that contains the corresponding block *m* (which may

correspond to for example, from 12 345bp to 24 568bp of chromosome 3) for the patient *n*.

Supplementary Figure 2. Data analysis flowchart

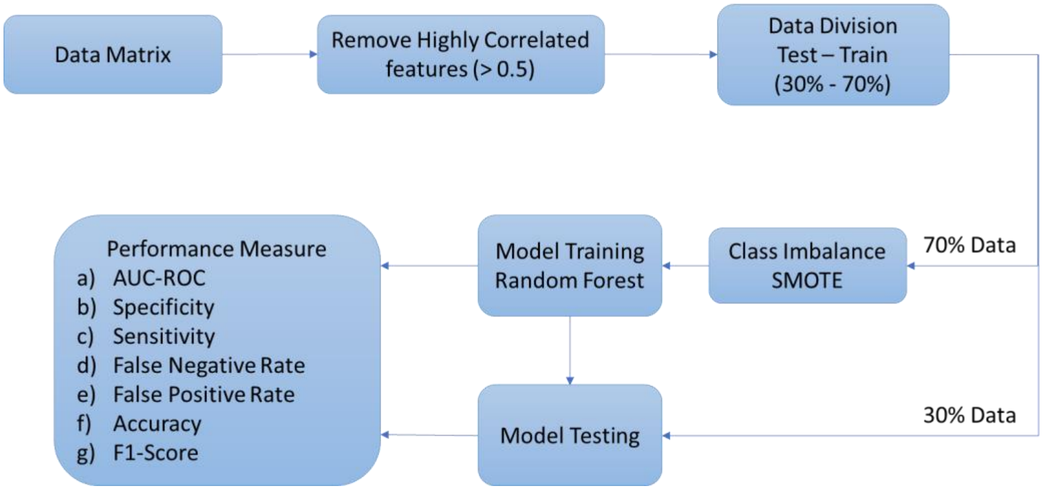

Supplementary Figure 3. Copy number aberrations for the (A) FHR (B) GHR (C) SR group

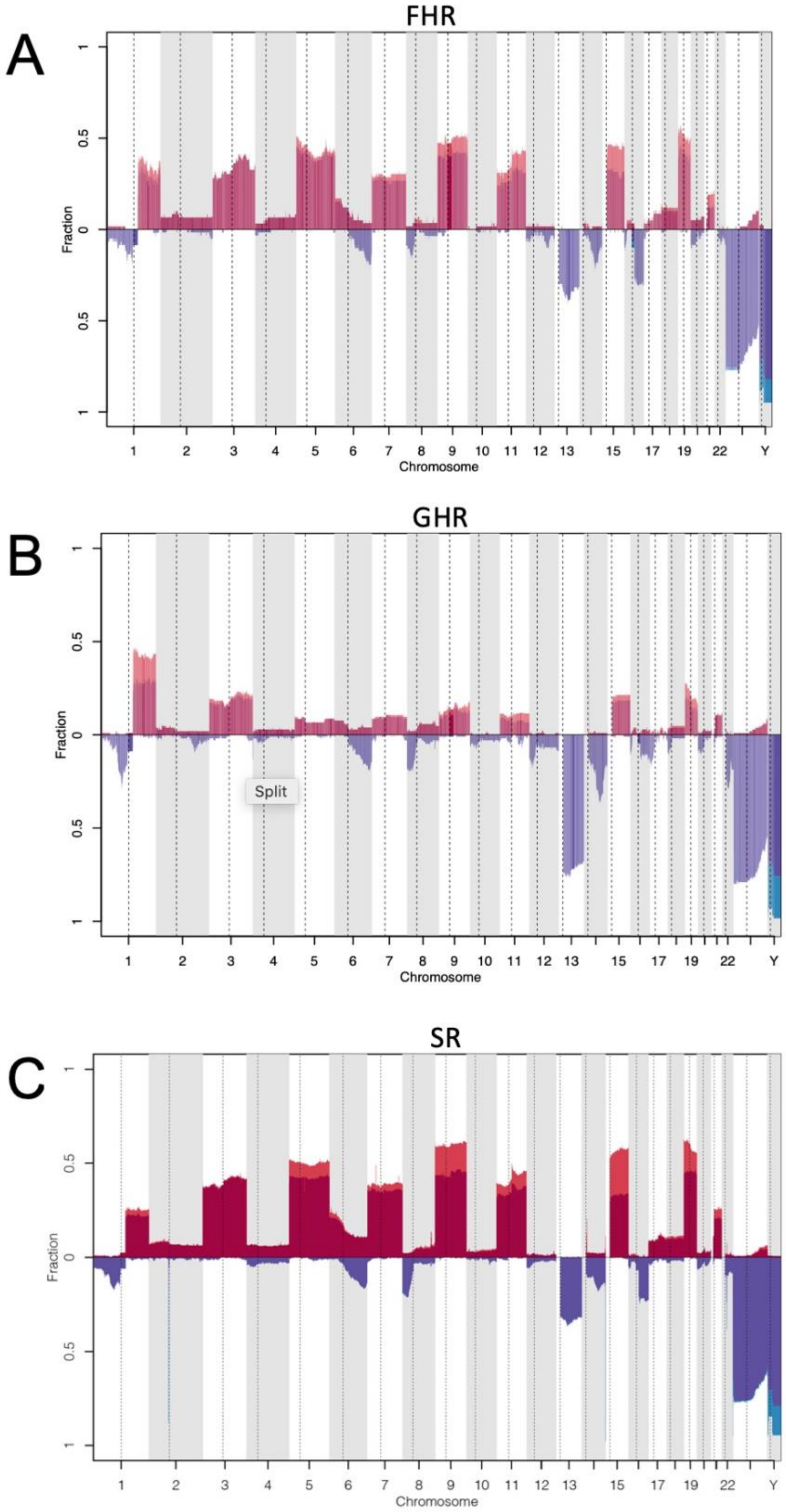

Supplementary Figure 4. Chromosomal instability index (CIN) among FHR, GHR, SR groups

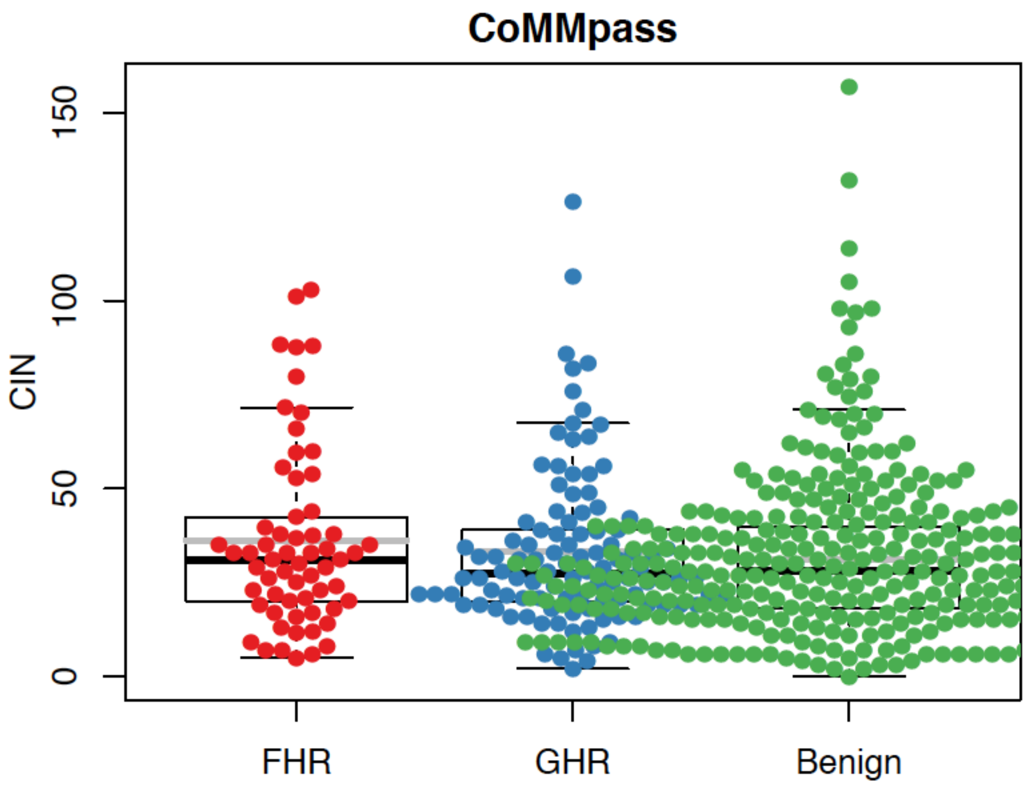

Supplementary Figure 5. Receiver Operating Characteristic (ROC) curve of a combination of mutation matrix, gene expression profiles and clinical parameters

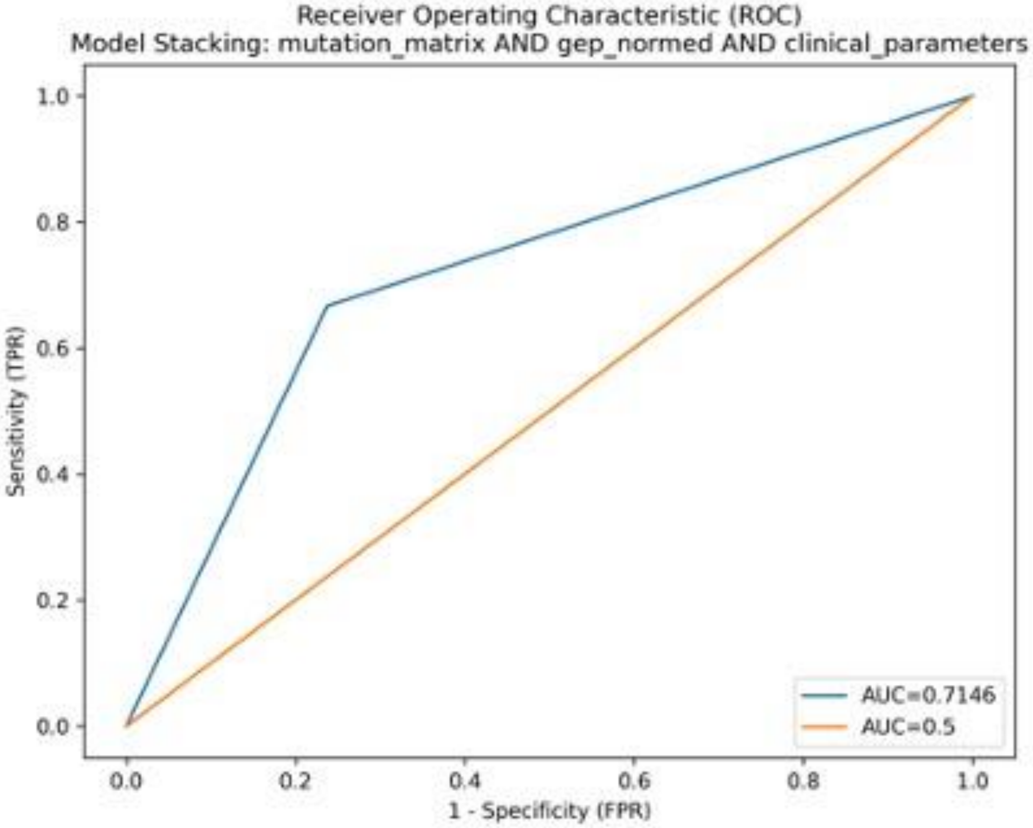

Supplementary Tables

Supplementary Table 1. Six generated Dataset used for the FHR predictive model

S No.	Name of Datasets	Description
1	cna_by_gene_reduced	This is the CNA status for each of 13 155 genes that harbored at least one CNA event. The CNA events (-2, -1, +1, +2) assigned according to the method described in Supplementary Figure 1 were used. When multiple CNA statuses appeared in a gene, the CNA status of the dominant span was selected. All genomic loci and gene locations were based on genome version hg19/GRCh37.
2	cna_by_arm	This is the CNA status for each of 44 chromosomal p or q-arms. As in the case of CNA by gene reduced, the

		CNA status of dominant span was selected when multiple CNA statuses appeared in a chromosomal arm.
3	mutation_count_by_arm	This is the number of all NS mutations for each of 44 chromosomal p or q-arms. It is also based on information retrieved from the file MMRF_CoMMpass_IA13a_All_Canonical_NS_Variants.txt.gz. All genomic locus information is based on genome version hg19/GRCh37.
4	mutation_matrix	This is the number of mutations for each of 15 633 genes that harbored at least one non-synonymous (NS) mutation. It is based on information retrieved from the file MMRF_CoMMpass_IA13a_All_Canonical_NS_Variants.txt.gz.
5	gep_normed	This is the normalized gene expression data for 25 554 genes from RNA-seq. Each gene's expression

		profile was normalized against its median level.
6	clinical_parameters	Six parameters that are routinely measured in myeloma clinics. The six parameters are age, gender, Eastern Cooperative Oncology Group (ECOG), International Staging System (ISS), Creatinine, Proliferation index.

Supplementary Table 2. Results for individual models

**Model_Stack**	**False Negative Rate**	**False Positive Rate**	**AUC-ROC**	**Accuracy**	**Specificity**	**Sensitivity**	**F1 Score**	**MCC**
cna_by_arm	0.3333	0.6475	0.5096	0.3831	0.3525	0.6667	0.1739	0.0119
cna_by_gene_reduced	0.0667	0.7842	0.5746	0.2857	0.2158	0.9333	0.2029	0.1103
mutation_matrix	0.1333	0.4748	0.6959	0.5584	0.5252	0.8667	0.2766	0.2324
mutation_count_arm	0.2667	0.5468	0.5933	0.4805	0.4532	0.7333	0.2157	0.1116
gep_normed	0.0667	0.7986	0.5674	0.2727	0.2014	0.9333	0.2000	0.1022
clinical_parameters	0.2000	0.5683	0.6158	0.4675	0.4317	0.8000	0.2264	0.1397

Supplementary Table 3. Results for Stacked Models

Model_Stack	False Negative Rate	False Positive Rate	AUC-ROC	Accuracy	Specificity	Sensitivity	F1 Score	MCC
gep_normed AND mutation_matrix	0.1333	0.3741	0.7463	0.6494	0.6259	0.8667	0.3250	0.2957
cna_by_gene_reduced AND mutation_matrix	0.2000	0.3669	0.7165	0.6494	0.6331	0.8000	0.3077	0.2612
**mutation_matrix AND gep_normed AND clinical_parameters**	**0.3333**	**0.2374**	**0.7146**	**0.7532**	**0.7626**	**0.6667**	**0.3448**	**0.2837**
**cna_by_gene_reduced AND mutation_matrix AND gep_normed**	**0.2000**	**0.2950**	**0.7525**	**0.7143**	**0.7050**	**0.8000**	**0.3529**	**0.3152**
cna_by_gene_reduced AND mutation_matrix AND gep_normed AND clinical_parameters	0.4000	0.2014	0.6993	0.7792	0.7986	0.6000	0.3462	0.2766

Supplementary Files

Supplementary File 1 (Features.zip, which consists of 6 csv files)

- Features used to build a predictive model to identify FHR patients

Supplementary File 2 (DEG_SAM.txt)

- The differential genes from SAM (SamFDR and SamD) to evaluate DEGs**.** P.fhr and abs(P.fhr)) = Results from Wilcoxon's rank sum test between FHR and SR; P.ghr and abs(P.ghr)) = Results from Wilcoxon's rank sum test between GHR and SR. P-values P.fhr and P.ghr contains negative values, which indicate expression levels in FHR (or GHR) is lower than those in SR.

Supplementary File 3 (FHR_Top200_Strict_David.txt)

- Functional annotations using DAVID for the top 200 genes from the comparison of FHR and SR groups

Supplementary File 4 (GHR_DEG_Top499_David_By_PSAM.txt)

- The top 499 genes from the comparison of GHR and SR

Supplementary File 5 (GSEA_FHR_vs_Benign_H_for_Fhr.tsv)

- Hallmark gene sets enriched towards FHR group from comparison between FHR and SR groups

Supplementary File 6 (GSEA_GHR_vs_Benign_H_for_Ghr.tsv)

- Hallmark gene sets enriched towards GHR group from comparison between GHR and SR groups
